# Scenario-Related Anxiety, Self-Reported Critical Incidents, and Burnout Among Anesthesia Professionals: A Cross-Sectional Survey

**DOI:** 10.3390/healthcare14172815

**Published:** 2026-09-02

**Authors:** Anıl Onur, Asiye Demirel

**Affiliations:** Department of Anesthesiology and Reanimation, University of Health Sciences, Bursa Yüksek İhtisas Training and Research Hospital, 16300 Bursa, Türkiye; dr.asiyedemirel@hotmail.com

**Keywords:** anesthesiology, patient safety, fear, anxiety, adverse events, second victim, burnout, simulation training, anesthesia technicians

## Abstract

**Highlights:**

**What are the main findings?**

- Pediatric complications, medication errors, and malignant hyperthermia received the highest scenario-specific anxiety ratings; difficult airway was most cited in a separate, non-equivalent incident item.
- All self-selected respondents reported a participant-defined career incident—a proportion that may be overestimated because of self-selection—while only 7.8% reported past-year simulation training.

**What are the implications of the main findings?**

- Scenario ratings and incident citations are separate descriptive profiles, not a direct comparison or estimates of event incidence or prevalence.
- The preliminary index and adjusted associations are hypothesis-generating; multisite studies using validated measures are required before clinical or policy use.

**Abstract:**

**Background/Objectives:** Scenario-specific anticipatory anxiety among anesthesia professionals is poorly characterized. We describe scenario ratings, career incidents, and associated factors. **Methods:** This cross-sectional survey (20 June–6 July 2026) included a self-selected convenience sample of 400 anesthesia professionals in Bursa, Türkiye. Participants rated 12 scenarios and reported incidents, post-event impact, burnout, and institutional preparedness. Multivariable regression examined the preliminary mean index. **Results:** An internal split-sample stability assessment—not independent validation—showed inadequate confirmatory fit; the 12-item mean was therefore treated as a preliminary descriptive index, not a validated scale. Ratings were highest for pediatric complications (7.01), medication errors (6.45), and malignant hyperthermia (6.15). Difficult airway was selected most often in a separate, non-equivalent incident item (95.8%); selections indicate citation salience, not prevalence. All respondents reported a participant-defined career incident. The invitation denominator was unknown; self-selection may have inflated the observed 100% incident-reporting proportion. Only 7.8% reported past-year simulation training. The exploratory model explained 10.9% of the index variance. Higher values were associated, after adjustment, with female sex (β = 0.55, 95% CI 0.15–0.96), faculty (β = 1.58, 95% CI 0.50–2.66) and specialist roles (β = 1.36, 95% CI 0.82–1.90) relative to technicians, and with a higher study-specific burnout rating (β = 0.20/point, 95% CI 0.09–0.31). Reporting ≥6 vs. <6 incidents was associated with a modestly lower value (β = −0.64, 95% CI −1.20 to −0.09), without a monotonic trend. **Conclusions:** The findings are hypothesis-generating. Selection, reverse causation, measurement error, untested cross-role measurement invariance, common method covariance, multiplicity, and unexplained variance limit interpretation. Multisite studies using validated measures are required before application.

## 1. Introduction

Anesthesia practice requires rapid decision-making during uncommon but potentially catastrophic events. Life-threatening complications such as failed airway management, intraoperative cardiac arrest, and malignant hyperthermia remain part of clinical practice and may impose a considerable cognitive and emotional burden on anesthesia professionals [1,2]. In the Fourth National Audit Project, severe airway complications were associated with substantial morbidity and mortality, and care was judged poor in three-quarters of assessable anesthesia cases [3].

Beyond their immediate clinical consequences, adverse events also affect the professionals involved in them. The term “second victim” describes clinicians who experience psychological distress, including guilt, anxiety, intrusive rumination, and loss of professional confidence, after patient safety incidents [4]. Recovery has been described as following a recognizable trajectory, and clinicians who are not adequately supported may develop lasting distress, defensive practice, or intention to leave the profession [5,6]. Surveys of physicians have reported anxiety about future errors, sleep disturbance, and reduced professional confidence after serious events [7]. The Second Victim Experience and Support Tool and its validated Turkish version provide standardized approaches to assessing these effects and available support resources [8,9]; a recent study in Turkish anesthesia and intensive care settings reported organizational support gaps [10].

Anesthesia professionals also face a substantial burnout burden. In a national survey of anesthesiologists in the United States, more than half of respondents were at high risk of burnout [11]. Burnout is commonly described through emotional exhaustion, depersonalization, and reduced personal accomplishment [12]. Some categorically anchored single-item measures have demonstrated validity against longer instruments [13], but that evidence does not directly validate the study-specific 0–10 item used here. A meta-analysis has also linked physician burnout with self-reported medical errors, although the underlying evidence was observational and self-reported [14].

Walsh and Beatty quantified anesthesiologists’ ratings of perceived severity and anxiety across 25 predefined clinical situations arising during anesthesia [15]. A recent survey of Jordanian anesthesiologists described specific perioperative concerns, with ventilation and intubation difficulty, hypoxia, and death among the prominent concerns [16]. Lapa et al. developed and psychometrically evaluated an anesthesia-specific stressor questionnaire whose clinical stress domain included anticipated difficult intubation; its domain scores were associated with burnout and other psychological outcomes [17]. Together, these studies establish important precedents. Evidence remains limited, however, on how multi-scenario anticipatory anxiety ratings relate to self-reported career-long critical incidents, post-event impact, and burnout, particularly in samples spanning physicians and anesthesia technicians and alongside perceived institutional preparedness. Whether greater self-reported career-long critical incident experience is associated with scenario-related anxiety remains insufficiently characterized. The scenarios include uncommon or rare high-acuity events as well as medication errors [18,19,20]. Simulation-based education can improve knowledge and technical skills in anesthesiology and human factor skills in healthcare teams, although evidence on patient outcomes is more limited [21,22].

Thus, this study aimed to (i) identify the complication scenarios that generate the highest anxiety among anesthesia professionals and conduct a preliminary psychometric evaluation of a newly developed 12-scenario anxiety item set; (ii) describe critical incident experiences, post-event psychological impact, burnout, and institutional preparedness; and (iii) explore demographic and professional factors associated with the preliminary mean scenario-specific anxiety index after multivariable adjustment.

## 2. Materials and Methods

### 2.1. Study Design and Setting

This cross-sectional, self-administered online survey was conducted among anesthesia professionals working in hospitals in Bursa Province, Türkiye, between 20 June and 6 July 2026. The study is reported in accordance with the STROBE statement for observational studies [23] and the CHERRIES checklist for internet-based surveys [24] (Supplementary Materials).

### 2.2. Participants and Recruitment

Eligible participants were anesthesiology and reanimation faculty members, specialists, residents, and anesthesia technicians/technologists aged 18–65 years who worked in anesthesia services in hospitals in Bursa Province. Individuals working outside anesthesia or outside Bursa Province were excluded. Consent was an ethical and procedural requirement, not an eligibility criterion (Section 2.4). In Türkiye, anesthesia technicians/technologists are regulated healthcare personnel who, under an anesthesiologist’s supervision and responsibility, undertake specified tasks related to airway management, medication administration, and the safe initiation, maintenance, and termination of anesthesia [25]. All displayed analytic items were required, so submitted questionnaires were complete and none were excluded for missing data. The Google Forms link was distributed through professional WhatsApp groups. The number of eligible clinicians who received or viewed the invitation was unknown; consequently, a conventional response rate could not be calculated. Duplicate participation was limited to one response per signed-in account. Sign-in served only to limit duplicate participation; no email address or account identifier was collected or accessible to the investigators. Before data collection, a target of 400 participants was specified in the ethics submission and study protocol; this target was achieved. The achieved sample permitted two random, non-overlapping internal subsamples of n = 200 for the factor structure stability assessment and was used for the planned multivariable modeling. With N = 400, a sensitivity calculation indicated approximately 81% power (two-sided α = 0.05) to detect an individual predictor effect of f^2^ = 0.02 in the primary regression model with seven non-intercept predictor terms. Because no directly comparable prior estimate was available, f^2^ = 0.02 was selected as the conventional benchmark for a small incremental effect in multiple regression. This value was used as a sensitivity reference for the detectable effect size rather than as a study-specific threshold of clinical importance.

### 2.3. Instrument

The self-administered questionnaire comprised 52 response variables in six sections: (A) sociodemographic and professional characteristics (8); (B) scenario-specific anxiety (17), including twelve 0–10 ratings, three ranked selections, and two selected anxiety sources; (C) critical incident experience (7); (D) post-event psychological impact (9), which was conceptually informed by the second victim literature and SVEST framework [8,9]; (E) burnout and work stress (6), including a four-item core, a separately analyzed work-meaningfulness item, and a study-specific 0–10 overall burnout rating; and (F) institutional preparedness (5). The 12 scenarios were developed by the investigators based on their clinical experience and a review of the relevant anesthesia complication literature. The draft items were informally reviewed by anesthesiologists for clinical relevance and clarity and were pilot-tested before data collection. Although some categorically anchored single-item burnout measures have demonstrated validity [13], the present 0–10 item was not a validated diagnostic measure; only within-sample convergence with the four-item core was examined. The mean of the investigator-developed 12-item scenario set was treated as a preliminary descriptive index, not as a validated psychometric or clinical scale; it had no standardized cutoff and was not designed for diagnosis, individual clinician classification, or clinical decision-making. The full questionnaire is provided in the Supplementary Materials (Questionnaire S1, English translation; Questionnaire S2, original Turkish form).

### 2.4. Ethics

The study was approved by the Medical Sciences Ethics Committee of the University of Health Sciences, Bursa Yüksek İhtisas Training and Research Hospital (protocol code 2024-TBEK 2026/06-27; 10 June 2026) and conducted in accordance with the Declaration of Helsinki. Participation was voluntary. The responses were anonymous to the research team: no directly identifying information was collected, and account identifiers used by the platform to limit duplicate submissions were not available to the investigators. Before beginning the first questionnaire section (Section A), participants had to select “Yes” on an electronic information-and-consent screen; selecting “No” ended the form.

### 2.5. Statistical Analysis

Categorical variables are presented as frequencies and percentages and continuous variables as mean ± standard deviation (SD) and median with interquartile range (IQR). The 0–10 scenario ratings were treated as continuous, and psychometric analyses used the Pearson correlation matrix. Internal consistency was assessed with Cronbach’s α, corrected item–total correlations, and McDonald’s ω total. Sampling adequacy was evaluated with the Kaiser–Meyer–Olkin measure and Bartlett’s test; exploratory factor analysis used minimum residual (MINRES) extraction with promax rotation. Factor retention considered eigenvalues > 1 and Horn’s parallel analysis (95th percentile from 200 random datasets). A one-factor confirmatory model was fitted for comparison. Because only two first-order factors were retained, the higher-order general factor loadings in a Schmid–Leiman decomposition are not uniquely identified. Sensitivity analyses examined equal loading and each first-order factor anchored in turn; ω hierarchical and explained common variance were therefore summarized as constraint-dependent ranges rather than unique point estimates. Confidence intervals used 1000 nonparametric bootstrap resamples with the full factor solution re-estimated in each resample.

Composite scores were calculated as item means: the twelve-item mean scenario-specific anxiety index (0–10), which was treated only as a preliminary descriptive index and prespecified as the primary outcome; a study-specific six-item post-event distress composite; and a study-specific four-item burnout core. Two factor-derived item means were retained only as post hoc, highly exploratory supplementary summaries and not as validated subscales. Because composite scores were non-normally distributed (Shapiro–Wilk *p* < 0.05), group comparisons used Kruskal–Wallis tests with Bonferroni-corrected pairwise Mann–Whitney U tests or Mann–Whitney U tests for two groups; correlations used Spearman’s rho, except that Pearson correlations were used for the within-item-set support/distress coherence checks reported in Section 3.5. Effect sizes were η^2^H for Kruskal–Wallis tests and r for Mann–Whitney U tests. Factor structure stability was assessed internally by exploratory factor analysis in one random half (n = 200) and maximum-likelihood CFA in the other. Because the full sample had already informed model specification, this was an internal stability check, not independent confirmation. Fit was summarized with CFI, TLI, and RMSEA. Formal multi-group measurement-invariance testing was not undertaken because the baseline two-factor CFA showed inadequate fit and the faculty subgroup was small (n = 30); together, these conditions did not support a sufficiently stable configural model as a prerequisite for metric and scalar invariance testing.

Adjusted associations with the preliminary mean index were explored using least-squares regression with heteroscedasticity-robust HC3 standard errors. Covariates were sex, professional role (reference: technician), years of experience, the questionnaire-defined ≥6 vs. <6 incident contrast, and the study-specific overall burnout rating. The ≥6 category was specified in the questionnaire before data collection as the highest top-coded incident count response; however, its use as a binary ≥6 vs. <6 predictor was not prespecified as a clinical threshold and was examined as an exploratory contrast. Diagnostics included variance inflation factors, studentized residuals, leverage, Cook’s distance, quadratic term checks, and refitting after influential observations were excluded; R^2^ and adjusted R^2^ are reported. Exploratory sensitivity analyses comprised an above-median logistic model, ordinal coding of the four incident count categories, scenario-matched comparisons, person-level fear–training concordance, and extended models including institution type, weekly caseload, and main field. Bonferroni adjustment was applied within specified comparison families. As a multiplicity sensitivity analysis, the Benjamini–Hochberg false discovery rate procedure was applied to the six raw *p* values from the scenario-matched comparisons. No correction was applied across the full study, so residual study-wide Type I error inflation remains possible. The preliminary mean index was the primary outcome; all other comparisons were hypothesis-generating. Two-sided *p* < 0.05 was used.

The analyses reported in the original submission were performed in Python 3.10 (pandas 2.3.3, SciPy 1.15.3, statsmodels 0.14.6, factor-analyzer 0.5.1, and semopy 2.3.11). During revision, the principal descriptive, regression, and psychometric results were reproduced in Python 3.12.13 using the same package versions, together with pyreadstat 1.3.6, NumPy 2.4.6, and scikit-learn 1.7.2. A fixed seed of 42 was used for the internal split-sample allocation and nonparametric bootstrap resampling. During initial manuscript preparation, Claude Fable 5 (Anthropic, San Francisco, CA, USA; accessed June–July 2026) assisted with language editing and statistical code checking. During revision, Codex (CLI version 0.149.0-alpha.4.1; model gpt-5.6-sol; OpenAI, San Francisco, CA, USA; accessed August 2026) assisted with additional computational reproducibility checks, source-supported language editing, and document preparation. Only a de-identified dataset was processed. The authors independently reviewed the code, numerical outputs, sources, and interpretations and take full responsibility for the work.

## 3. Results

### 3.1. Participant Characteristics

Four hundred anesthesia professionals participated (mean age 36.5 ± 9.6 years; 53.5% female). Technicians constituted 36.2% of the sample, residents 29.2%, specialists 27.0%, and faculty members 7.5%. Median professional experience was 10 years (IQR 4–17) and median weekly caseload was 22 (IQR 20–30); 71.5% worked in training and research hospitals and 84.8% in mixed/general practice (Table 1).

### 3.2. Preliminary Psychometric Evaluation of the Scenario-Specific Anxiety Item Set

The twelve scenario-specific anxiety items showed high internal consistency (Cronbach’s α = 0.911), with corrected item–total correlations of 0.49–0.79; removing any item did not improve α. Sampling adequacy was high (KMO = 0.901; Bartlett’s χ^2^(66) = 2651.0, *p* < 0.001). Exploratory factor analysis yielded two factors (eigenvalues 6.14 and 1.28); the variance proportions reported for the rotated solution were 37.7% and 17.1% (cumulative 54.9%). Eight items loaded primarily on Factor 1 and four on Factor 2; intraoperative awareness loaded weakly on both (0.24/0.32). Parallel analysis only marginally supported the second factor (observed eigenvalue 1.28 vs. 95th-percentile random threshold 1.26), and the promax factor correlation was 0.67 (Table 2). Because confirmatory support was inadequate, the factors were not assigned substantive domain interpretations in the main analysis.

Because the full sample had already informed model specification, the internal split-sample assessment was a stability check, not an independent validation. Exploratory factor analysis in one random half (n = 200; KMO = 0.887; eigenvalues 5.98 and 1.33) yielded the same item assignment, although awareness again loaded weakly and nearly equally (0.26/0.28). CFA in the other half (n = 200) showed inadequate fit for the proposed two-factor model (χ^2^(53) = 207.2; CFI = 0.887; TLI = 0.859; and RMSEA = 0.121). A one-factor model fitted still worse (χ^2^(54) = 281.2; CFI = 0.833; TLI = 0.796; and RMSEA = 0.145).

McDonald’s ω total was 0.93 (95% CI 0.92–0.94). With only two first-order factors, however, the hierarchical general factor decomposition was underidentified: across the three identification constraints examined, ω hierarchical ranged from 0.65 to 0.87, whereas explained common variance ranged from 0.62 to 0.83. No single ω hierarchical or explained common variance estimate was therefore interpreted, and ω total was not treated as evidence of unidimensionality or clinical validity. Configural, metric, and scalar invariance were not tested across faculty, specialists, residents, and technicians; observed role differences may reflect item interpretation differences as well as reported anxiety. The twelve-item mean was retained only as a preliminary descriptive index, not a validated scale, and was not intended for diagnosis, individual classification, or clinical decisions. Factor-derived item means were confined to a post hoc, highly exploratory supplementary analysis.

### 3.3. Scenario-Specific Anxiety

**Figure 1:**
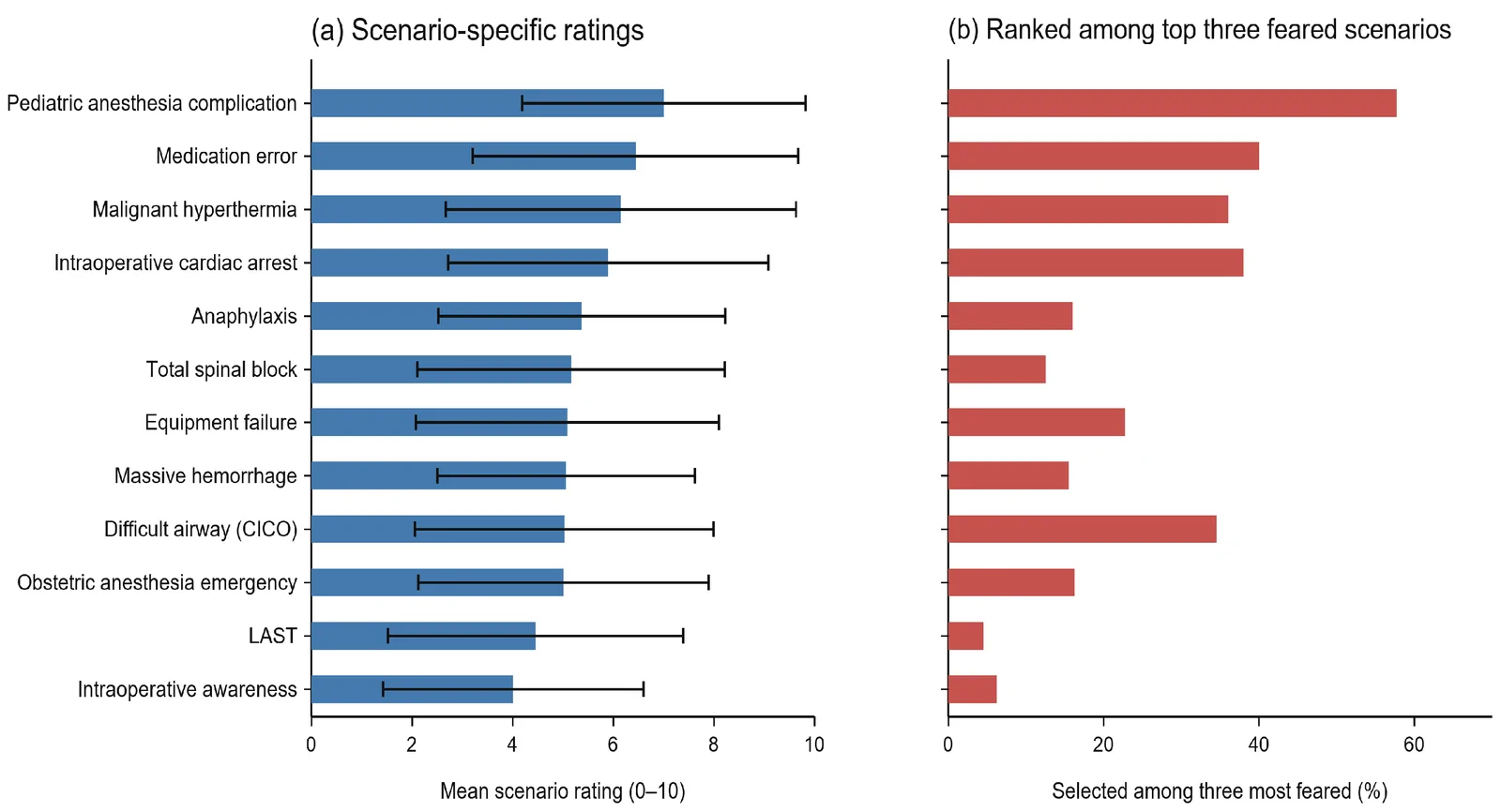
Descriptive scenario-related anxiety findings among anesthesia professionals (N = 400). (**a**) Mean study-specific scenario ratings (0–10; error bars: SD). (**b**) Percentage ranking each scenario among the three most feared scenarios from 12 options. The panels summarize two response formats within the same scenario set and do not represent clinical anxiety or objective event risk.

The highest scenario ratings were observed for pediatric anesthesia complications (7.01 ± 2.82), followed by medication errors (6.45 ± 3.24), malignant hyperthermia (6.15 ± 3.48), and intraoperative cardiac arrest (5.90 ± 3.18), whereas intraoperative awareness (4.01 ± 2.59) and LAST (4.46 ± 2.94) received the lowest ratings (Table 3, Figure 1). In an exploratory cross-scenario comparison, the ranking task yielded a similar ordering (Spearman’s rho = 0.80 between the mean scenario ratings and the top-three selection frequencies across the twelve scenarios, *p* = 0.002): 57.8% of participants placed pediatric complications among their three most feared scenarios, followed by medication error (40.0%), cardiac arrest (38.0%), malignant hyperthermia (36.0%), and difficult airway (34.5%). The most frequently selected sources of anxiety were the possibility of permanent patient harm or death (53.0%) and medicolegal liability (43.2%).

### 3.4. Critical Incident Experience

Within this self-selected convenience sample, all participants (100%) reported at least one participant-defined serious/critical career incident, and 81.2% selected the top-coded category of six or more. A high within-sample proportion is clinically plausible given the career-long recall window, broad participant-defined wording, and inclusion of common challenges such as difficult airway. Nevertheless, the unknown invitation denominator and self-selection may have increased the observed proportion; it is not an incidence or population prevalence estimate. In a separate fixed-choice item, each participant selected two serious event types from six specified categories plus “Other”. Difficult airway (95.8%), cardiac arrest (55.8%), and medication error (31.0%) were cited most often. Two respondents (0.5%) selected “Other” without entering a specification. Permanent patient harm was reported by 8.2%. Only 58.8% formally reported their most serious event, and 12.2% stated that no reporting system existed; among those whose institution had a system, 67.0% (235/351) formally reported the event. In addition, 66.2% indicated a positive influence on clinical decision-making (Table 4). In an exploratory scenario-matched analysis, participants citing cardiac arrest reported higher ratings for that scenario than those who did not (median 7 vs. 5, Bonferroni-corrected *p* = 0.012, r = 0.15); the other five matchable scenarios did not differ. A Benjamini–Hochberg false discovery rate sensitivity analysis produced the same interpretation: only the cardiac arrest comparison remained significant (q = 0.012; Table S8).

### 3.5. Post-Event Psychological Impact

The six distress items formed an internally consistent, study-specific composite (α = 0.877). The two support items did not cohere positively with the six distress items (correlations with the six-item distress mean: r = 0.03 for collegial support and r = −0.18 for institutional support) and were essentially uncorrelated with each other (r = 0.02); they were therefore analyzed separately rather than included in the distress composite. The distress statement with the highest endorsement was fear of recurrence (30.5% agreement; mean 3.05 ± 0.95), followed by intrusive rumination (24.5%), and 15.0% of participants had considered leaving the profession because of the event. Perceived support from colleagues (69.2% agreement; 3.82 ± 0.99) was substantially higher than perceived institutional support (27.0%; 2.92 ± 0.98), and 88.2% of participants had been able to share the event with colleagues (Table 4).

### 3.6. Burnout and Work Stress

The mean self-rated overall burnout score was 5.6 ± 1.8 (median 6), and 33.2% of participants scored 7 or higher (a descriptive, study-specific categorization rather than a validated clinical threshold). The single-item burnout score correlated strongly with the four-item burnout core (rho = 0.66, *p* < 0.001) and inversely with meaningfulness of work (rho = −0.53, *p* < 0.001), showing within-sample convergence but not establishing construct validity, equivalence to a validated inventory, or a clinical cutoff. Nevertheless, 67.8% of participants reported often or always finding their work meaningful (Table 4).

### 3.7. Institutional Preparedness

Although 75.8% of participants reported that a standardized difficult airway protocol was available in their institution and 58.0% considered the incident reporting system functional, only 7.8% had received simulation-based emergency scenario training within the preceding year. The most frequently requested training domains were difficult airway management (60.0%), cardiopulmonary resuscitation (46.8%), and medication safety (29.5%), which partly paralleled the observed fear hierarchy. In an exploratory, hypothesis-generating person-level analysis, concordance was observed for three of the five matchable domains: participants who ranked difficult airway among their three most feared scenarios requested difficult airway training more often than those who did not (73.2% vs. 53.1%, Bonferroni-corrected *p* < 0.001), with similar patterns for malignant hyperthermia (32.6% vs. 12.1%, corrected *p* < 0.001) and medication safety (38.8% vs. 23.3%, corrected *p* = 0.007); no significant association was observed for cardiac arrest or anaphylaxis.

### 3.8. Factors Associated with Scenario Anxiety

**Figure 2:**
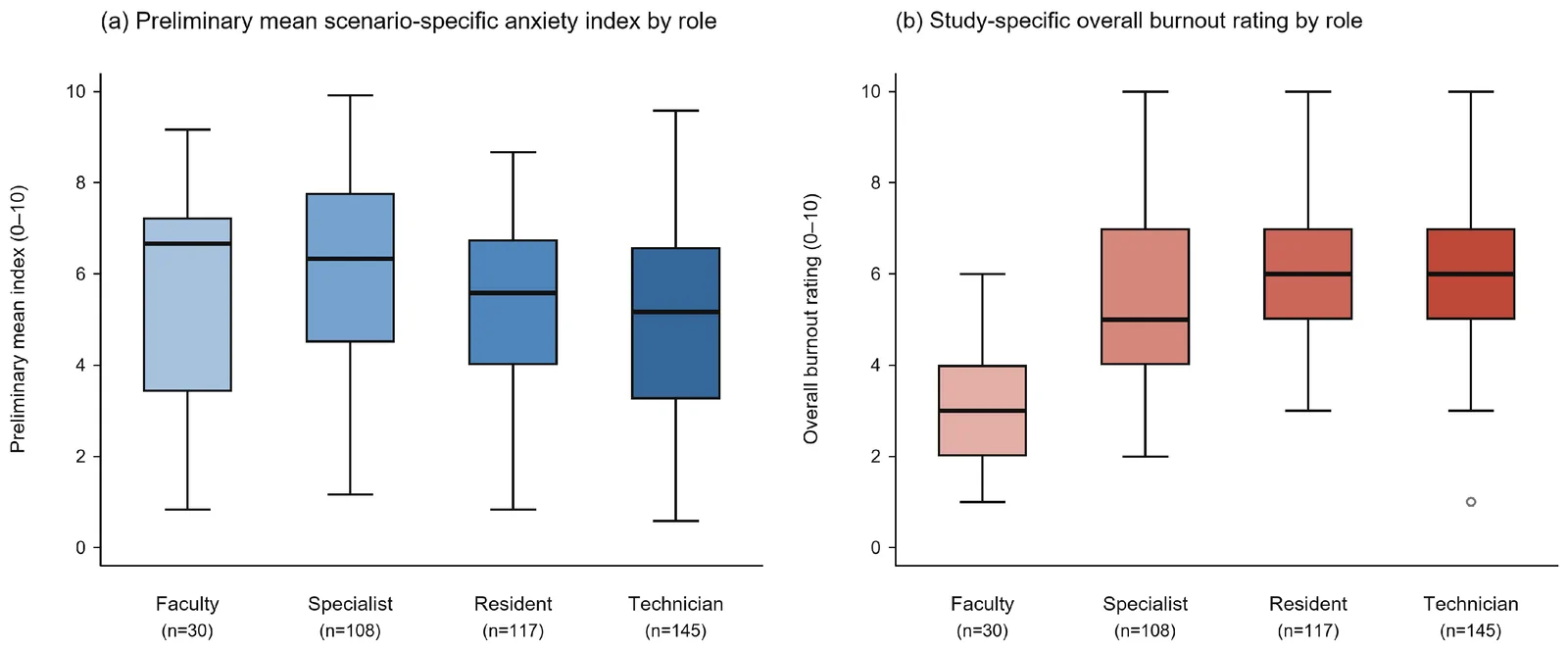
Distribution of (**a**) the preliminary mean scenario-specific anxiety index and (**b**) the study-specific overall burnout rating by professional role (N = 400). Boxes: median and interquartile range; whiskers: 1.5 × IQR; and circles: observations beyond the whiskers. Formal measurement invariance was not tested; between-role differences are descriptive comparisons of observed values, not validated latent construct differences. Color shades distinguish professional-role categories only and do not encode an additional variable.

Observed values of the preliminary mean scenario-specific anxiety index differed across professional roles (Kruskal–Wallis H = 16.94, *p* < 0.001); formal measurement invariance across the four roles was not tested. Post-event distress was highest among specialists (*p* < 0.001 vs. residents and technicians). Self-rated overall burnout also differed markedly across roles (H = 69.35, *p* < 0.001; medians: faculty 3, specialist 5, resident 6, and technician 6), with an inverse pattern for meaningfulness of work (Table 5, Figure 2). Role effects were largest for overall burnout (η^2^H = 0.168) and meaningfulness of work (η^2^H = 0.116) and small for the preliminary index (η^2^H = 0.035) and post-event distress (η^2^H = 0.048). Women reported higher preliminary mean index values (median 6.08 vs. 5.08, *p* < 0.001) and post-event distress (*p* = 0.047) than men (r = 0.17 and 0.10, respectively), whereas no sex difference was observed for burnout (*p* = 0.805). The preliminary index was not associated with age or experience, whereas burnout decreased (rho = −0.174, *p* < 0.001) and meaningfulness of work increased (rho = 0.117, *p* = 0.019) with increasing experience. The preliminary index correlated moderately with post-event distress (rho = 0.340, *p* < 0.001) and showed a very small association with burnout (rho = 0.098, *p* = 0.050); given its magnitude and the exploratory multiplicity, this finding was not interpreted substantively.

The primary exploratory continuous model explained 10.9% of the preliminary index variance (R^2^ = 0.109; adjusted R^2^ = 0.093), leaving most variation unexplained (Table 6). Higher values were associated after adjustment with female sex (β = 0.55, 95% CI 0.15–0.96), faculty (β = 1.58, 95% CI 0.50–2.66), and specialist roles (β = 1.36, 95% CI 0.82–1.90) relative to technicians, and the study-specific overall burnout rating (β = 0.20 per point, 95% CI 0.09–0.31). Reporting ≥6 rather than <6 incidents was associated with a lower value (β = −0.64, 95% CI −1.20 to −0.09). Resident role (β = 0.03, 95% CI −0.48 to 0.53) and years of experience (β = −0.03/year, 95% CI −0.06 to 0.01) were not associated after adjustment.

All VIFs were <1.9 (1.03–1.88); maximum Cook’s distance was 0.03, no studentized residual exceeded |3|, and excluding the five most influential observations produced similar estimates (≥6 incidents: β = −0.62, *p* = 0.028). Quadratic terms for experience and burnout were nonsignificant (*p* = 0.38 and 0.39). When incident count was modeled ordinally across its four categories, the linear trend was nonsignificant (β = −0.20/category, 95% CI −0.54 to 0.14, *p* = 0.251), and category means were non-monotonic. The association was therefore specific to the questionnaire-defined ≥6 vs. <6 contrast and should not be interpreted as a validated cutoff or a dose–response threshold.

The exploratory above-median logistic sensitivity model (Table S4) was directionally consistent but had modest discrimination (Nagelkerke R^2^ = 0.120; AUC = 0.670) and did not define a clinical threshold. The results were similar after adding the institution type, weekly caseload, and main field (Tables S5 and S6). In the extended continuous model, ≥6 incidents remained associated with a lower value (β = −0.65, 95% CI −1.21 to −0.08, *p* = 0.024); higher weekly caseload (β = −0.026/case, 95% CI −0.043 to −0.010) and private hospital employment (β = −1.17, 95% CI −2.22 to −0.12) were also associated with lower values. All coefficients and sensitivity models are hypothesis-generating, not estimates of clinical importance or prediction.

## 4. Discussion

Scenario-related anxiety is an understudied component of anesthesia practice. A Jordanian survey similarly identified ventilation/intubation difficulty and hypoxia among prominent concerns [16], although direct numerical comparison is inappropriate because its sampling, content, and response format differed. Pediatric complications, medication errors, and malignant hyperthermia received the highest ratings; difficult airway was cited most often in a separate incident-type item. These non-equivalent profiles cannot be interpreted as directly comparable measures or as evidence about the relation between scenario ratings and incident occurrence. The 100% career incident proportion arose within a self-selected sample and may be increased by selection, although a career-long window, broad participant-defined incident wording, and the inclusion of difficult airway make a high within-sample proportion clinically plausible. The primary model explained 10.9% of the variance in the preliminary index, and the split-sample procedure was an internal stability assessment rather than an independent validation.

The descriptive juxtaposition may generate hypotheses about controllability, familiarity, anticipated harm, and personal responsibility, but cannot compare ratings with event frequency. Scenario content was conceptually heterogeneous: the pediatric complication item was a broad, population-defined category; the malignant-hyperthermia item referred to a specific syndrome with rare clinical reactions [20]; and the CICO rating was narrower than the broad difficult airway incident category. The ≥6 vs. <6 contrast was modest (β = −0.64; approximately 0.3 SD), category means were non-monotonic, and the ordinal sensitivity analysis was null. The finding does not identify a mechanism; reverse causation, survivor or career-retention selection, differential recall, questionnaire-defined dichotomization, residual confounding, measurement error, and chance are also plausible. Lower self-reported ratings must not be equated with competence, preparedness, performance, or patient safety. The scenario-matched cardiac arrest result was one of many exploratory tests and requires independent replication.

Observed preliminary index values differed by professional role and sex. Faculty and specialists had higher adjusted values than technicians, and women had higher values than men. Configural, metric, and scalar invariance were not tested across the four roles [27]; these contrasts may reflect item interpretation as well as reported anxiety. Faculty participants showed the highest descriptive scenario-specific anxiety values but the lowest burnout ratings. This contrasting pattern may reflect greater responsibility for high-acuity decision-making and medicolegal exposure, which could increase anticipatory scenario anxiety, alongside greater professional autonomy, schedule control, accumulated experience, and selection or retention effects that may reduce burnout. These explanations remain speculative because the faculty subgroup was small (n = 30) and the cross-sectional design does not permit causal interpretation. Residents did not differ from technicians after adjustment despite higher descriptive burnout, underscoring that the study-specific constructs are not interchangeable.

Fear of recurrence was the most frequently endorsed post-event consequence (30.5%), while perceived collegial support (69.2%) exceeded institutional support (27.0%). Anesthesia-specific surveys from Belgium and Germany document second victim experiences and variation or limitations in support structures [28,29]. An international consensus paper provides a cross-professional definition of the phenomenon and emphasizes its organizational implications [30]. Direct numerical comparisons are inappropriate because definitions, measures, recall periods, and professional populations differ. The collegial–institutional gap is therefore a local service evaluation signal, not an estimate of comparative international performance.

Residents and technicians reported higher descriptive burnout and lower work meaningfulness than faculty, and burnout decreased weakly with experience. Contemporary surveys document a substantial anesthesia-workforce well-being burden in the United States [31] and China [32]. A separate survey of 1508 specialist anesthesiologist respondents from 32 European countries found widespread self-reported work-related fatigue and limited organizational support [33]. Their validated or differently structured instruments are not numerically interchangeable with our study-specific 0–10 item. The adjusted burnout coefficient is therefore an exploratory within-sample association, not a clinical effect estimate.

Only 7.8% of this local convenience sample reported simulation-based emergency training in the preceding year. European residency and Canadian academic institution surveys show that simulation access, frequency, resources, and implementation vary across systems [34,35]. Their sampling frames and definitions differ, so 7.8% is not a national estimate for Türkiye or a direct international comparison. Exploratory training-request patterns partly paralleled rankings, but this study did not test whether training changes anxiety, burnout, performance, or patient outcomes. Ratings and self-reported needs are only complementary local curriculum inputs alongside epidemiologic risk, observed performance, feasibility, and institutional priorities.

### Strengths and Limitations

A strength of this study is the inclusion of faculty, specialists, residents, and technicians, together with transparent reporting of a newly developed item set and robustness checks. Important limitations remain. Convenience recruitment through professional messaging groups provided no known invitation/view denominator and permitted self-selection; clinicians with salient or emotionally memorable events may have been more likely to participate. The observed proportion—100% of respondents reporting at least one participant-defined career incident—may therefore be inflated by self-selection and cannot be generalized as an incident rate or lifetime prevalence, despite the clinical plausibility of a high within-sample proportion under the broad career-long item. The study was conducted in one province, the incident term was not standardized, event timing was not recorded, and retrospective self-report is vulnerable to recall bias. The cross-sectional design precludes causal inference. Hospital identity was unavailable, so within-hospital clustering could not be modeled and some standard errors may be underestimated. Accordingly, the findings should not be interpreted as national estimates for Türkiye.

The mean of the 12 scenario ratings is a preliminary descriptive index, not a fully validated psychometric or clinical scale. High internal-consistency estimates (α and ω total) do not establish unidimensionality or validity; CFA fit was inadequate, and the two-factor hierarchical decomposition was identification-dependent. The index has no diagnostic cutoff and must not be used to diagnose or classify individual clinicians or guide clinical decisions. Conceptual heterogeneity further limits interpretation: items range from a broad population to a rare syndrome, a process error, and a narrowly defined crisis. Although the items underwent informal expert review and pilot testing, no structured content validity procedure, such as a Delphi process or a content validity index, was performed; therefore, the comprehensiveness and content validity of the scenario set remain unestablished. Measurement invariance was not tested across roles. Factor-derived item means are retained only in Table S7 as post hoc, highly exploratory summaries and not as validated subdomains.

Scenario ratings, post-event impact, burnout, support, and incident experience were collected from the same person at one time using study-specific self-reports. The burnout and distress composites are not substitutes for validated inventories. Outcome measurement error may reduce precision. Predictor measurement error or nonclassical error can attenuate or inflate coefficients and affect other covariate estimates [26]. No dedicated assessment of common method variance was performed. Common method covariance arising from current affect, response styles, recall, or consistency motives may also inflate or otherwise distort the burnout–index association [36,37]; its direction and magnitude cannot be determined here. No marker variable, latent common method factor, temporal separation, or independent data source was available. All adjusted findings require confirmation with validated, preferably temporally separated and objective or institutional measures.

Incident count was broad and top-coded, with 81.2% in the ≥6 category. The questionnaire-defined dichotomy did not show a monotonic dose–response; reverse causation, survivor/career-retention selection, differential recall, residual confounding, and chance remain plausible. Rankings (top three of 12) and incident citations (two of six specified categories plus Other) had non-equivalent content and rules; pediatric complications had no incident counterpart, and difficult airway wording differed. Incident percentages indicate citation salience only. Finally, many exploratory analyses were conducted. Although Bonferroni adjustment was used within selected families and a Benjamini–Hochberg false discovery rate sensitivity analysis was applied to the six scenario-matched comparisons, no multiplicity correction was applied across the full study; residual Type I error inflation is therefore possible. Factor-derived, scenario-matched, fear–training, logistic, and extended model results are hypothesis-generating.

## 5. Conclusions

In this self-selected convenience sample of 400 anesthesia professionals, pediatric complications, medication errors, and malignant hyperthermia received the highest scenario ratings, while difficult airway was most frequently cited in a separate incident-type item. The non-equivalent item sets cannot support a direct comparison or inferences about the relation between scenario ratings and incident occurrence, incidence, or prevalence. The primary exploratory model explained 10.9% of the variance in the preliminary index. Its ≥6 vs. <6 incident contrast was modest and did not extend to a monotonic dose–response pattern; reverse causation, survivor selection, measurement error, and residual confounding remain plausible. Respondent-reported gaps in recent simulation training and institutional post-event support identify areas for local evaluation, but this cross-sectional study did not test training or support interventions. The 12-item mean should be interpreted only as a preliminary descriptive index, and all adjusted associations require replication in multisite studies using refined, validated measures before clinical or policy application.

## Figures and Tables

**Table 1:** Sociodemographic and professional characteristics of participants (N = 400).

Characteristic	Value (N = 400)
Age (years), mean ± SD	36.5 ± 9.6
Age (years), median (IQR)	33 (29–43.25)
**Sex, n (%)**	
Female	214 (53.5)
Male	186 (46.5)
**Professional role, n (%)**	
Faculty member (academic anesthesiologist)	30 (7.5)
Specialist anesthesiologist	108 (27.0)
Resident (anesthesiology trainee)	117 (29.2)
Anesthesia technician	145 (36.2)
Professional experience (years), median (IQR)	10 (4–17)
**Institution type, n (%)**	
Training and research hospital	286 (71.5)
University hospital	50 (12.5)
State hospital	39 (9.8)
Private hospital	25 (6.2)
**Main field of practice, n (%)**	
Mixed/general practice	339 (84.8)
Cardiac anesthesia	29 (7.2)
Other subspecialty areas	32 (8.0)
Weekly anesthesia cases, median (IQR)	22 (20–30)

SD: standard deviation; IQR: interquartile range. No participant selected the “prefer not to say” option for sex.

**Table 2:** Item analysis and promax pattern coefficients of the scenario anxiety items (N = 400).

Scenario Item	Corrected Item–Total r	Factor 1 Loading	Factor 2 Loading	Assigned
Difficult airway/failed intubation (CICO)	0.65	**0.52**	0.22	F1
Intraoperative cardiac arrest	0.79	**0.82**	0.05	F1
Anaphylaxis	0.76	**0.75**	0.08	F1
Malignant hyperthermia	0.65	**1.04**	−0.36	F1
Intraoperative awareness	0.49	0.24	**0.32**	F2
Total spinal block	0.61	**0.65**	0.00	F1
Local anesthetic systemic toxicity (LAST)	0.71	**0.72**	0.05	F1
Massive hemorrhage/hemodynamic collapse	0.64	0.30	**0.46**	F2
Medication error (wrong drug/dose)	0.67	**0.67**	0.06	F1
Pediatric anesthesia complication	0.72	**0.60**	0.20	F1
Obstetric anesthesia emergency	0.57	−0.11	**0.90**	F2
Equipment failure	0.49	−0.15	**0.84**	F2

Bold values indicate the larger promax pattern coefficient. F1: Factor 1; F2: Factor 2; CICO: cannot intubate, cannot oxygenate; and LAST: local anesthetic systemic toxicity. In an oblique solution, a pattern coefficient above 1 does not by itself establish a Heywood case; no communality exceeded 1. The inadequate confirmatory fit precludes interpreting the factors as validated subscales or substantive domains.

**Table 3:** Scenario-specific ratings and fear rankings (N = 400).

Scenario	Mean ± SD	Median (IQR)	Ranked 1st, n	Top Three, n (%)
Pediatric anesthesia complication	7.01 ± 2.82	8 (5–10)	80	231 (57.8)
Medication error (wrong drug/dose)	6.45 ± 3.24	7 (4–9.25)	50	160 (40.0)
Malignant hyperthermia	6.15 ± 3.48	7 (3–10)	54	144 (36.0)
Intraoperative cardiac arrest	5.90 ± 3.18	6 (3–9)	61	152 (38.0)
Anaphylaxis	5.37 ± 2.86	5 (3–8)	10	64 (16.0)
Total spinal block	5.17 ± 3.05	5 (2.75–8)	13	50 (12.5)
Equipment failure	5.09 ± 3.01	5 (3–8)	37	91 (22.8)
Massive hemorrhage/hemodynamic collapse	5.06 ± 2.56	5 (3–7)	9	62 (15.5)
Difficult airway/failed intubation (CICO)	5.03 ± 2.97	5 (2–7.25)	60	138 (34.5)
Obstetric anesthesia emergency	5.01 ± 2.89	5 (2–7)	13	65 (16.2)
Local anesthetic systemic toxicity (LAST)	4.46 ± 2.94	4 (2–7)	3	18 (4.5)
Intraoperative awareness	4.01 ± 2.59	4 (2–5)	10	25 (6.2)

Scenarios ordered by descending mean rating. IQR: interquartile range. Top three: selected among the three most feared scenarios from the same 12-scenario item set. CICO: cannot intubate, cannot oxygenate; and LAST: local anesthetic systemic toxicity. Ratings and rankings are descriptive self-reports and do not represent a validated clinical scale or objective risk.

**Table 4:** Critical incident experience, post-event psychological impact, and burnout (N = 400).

Variable	Value or Mean ± SD	n (%)
**Critical incident experience †**		
Experienced ≥ 1 serious critical incident, n (%)	400 (100.0)	
Experienced ≥ 6 incidents, n (%)	325 (81.2)	
Serious incident type reported: difficult airway, n (%)	383 (95.8)	
Serious incident type reported: cardiac arrest, n (%)	223 (55.8)	
Serious incident type reported: medication error, n (%)	124 (31.0)	
Serious incident type reported: anaphylaxis, n (%)	44 (11.0)	
Serious incident type reported: awareness, n (%)	16 (4.0)	
Serious incident type reported: malignant hyperthermia, n (%)	8 (2.0)	
Serious incident type reported: Other (unspecified), n (%) †	2 (0.5)	
Permanent patient harm, n (%)	33 (8.2)	
Event formally reported, n (%)	235 (58.8)	
No reporting system in institution, n (%)	49 (12.2)	
Positive influence on clinical decision-making, n (%)	265 (66.2)	
**Post-event psychological impact (1–5)**	**Mean ± SD**	**Agree, n (%) ‡**
Difficulty putting the event out of one’s mind	2.80 ± 1.09	98 (24.5)
Sleep or appetite disturbance	1.98 ± 0.85	20 (5.0)
Feelings of guilt or inadequacy	2.64 ± 0.86	59 (14.8)
Fear of recurrence of a similar event	3.05 ± 0.95	122 (30.5)
Shaken professional confidence	2.39 ± 0.94	50 (12.5)
Considered leaving the profession	2.34 ± 1.08	60 (15.0)
Adequate support from colleagues	3.82 ± 0.99	277 (69.2)
Adequate support from the institution	2.92 ± 0.98	108 (27.0)
Able to share the event with colleagues, n (%)	353 (88.2)	
**Burnout and work stress (1–5)**	**Mean ± SD**	**Often/always, n (%)**
Feeling emotionally exhausted at work	2.99 ± 0.88	107 (26.8)
Feeling overwhelmed by workload	2.92 ± 0.96	112 (28.0)
Difficulty going to work in the morning	2.44 ± 0.94	41 (10.2)
Work-related anxiety/tension	3.00 ± 0.98	123 (30.8)
Finding one’s work meaningful	3.77 ± 0.90	271 (67.8)
Overall burnout (0–10), mean ± SD; median (IQR)	5.6 ± 1.8	6 (4–7)
Overall burnout ≥ 7, n (%)	133 (33.2)	

The 100.0% value for at least one participant-defined career incident is a respondent proportion in this self-selected convenience sample; it may be overestimated because of self-selection and is not an incidence or population prevalence estimate. † Each participant selected exactly two serious incident types from six specified categories plus an “Other” option; participant-level percentages therefore sum to approximately 200% because of rounding. Two respondents selected “Other” without a free-text specification. Percentages indicate citation salience in this self-selected sample, not event frequency, incidence, or lifetime prevalence. The incident item was not equivalent to the top-three ranking from 12 scenarios, and pediatric complications had no incident-type counterpart. ‡ Agree: score 4–5. Post-event items were rated 1 (strongly disagree) to 5 (strongly agree); burnout items were rated 1 (never) to 5 (always). SD: standard deviation; IQR: interquartile range.

**Table 5:** Composite scores by professional role: median (IQR) and Kruskal–Wallis results.

Variable	Faculty (n = 30)	Specialist (n = 108)	Resident (n = 117)	Technician (n = 145)	*p* *
Preliminary mean scenario-specific anxiety index (0–10)	6.67 (3.42–7.23)	6.33 (4.50–7.77)	5.58 (4.00–6.75)	5.17 (3.25–6.58)	**<0.001**
Post-event distress (1–5)	2.58 (2.04–2.83)	2.83 (2.33–3.21)	2.33 (1.83–2.83)	2.33 (1.83–2.83)	**<0.001**
Burnout core items (1–5)	2.25 (1.75–2.50)	3.00 (2.50–3.50)	2.75 (2.50–3.50)	2.75 (2.25–3.25)	**<0.001**
Overall burnout (0–10)	3.00 (2.00–4.00)	5.00 (4.00–7.00)	6.00 (5.00–7.00)	6.00 (5.00–7.00)	**<0.001**
Meaningfulness of work (1–5)	5.00 (4.00–5.00)	4.00 (4.00–5.00)	4.00 (3.00–4.00)	4.00 (3.00–4.00)	**<0.001**

* Kruskal–Wallis test. Pairwise comparisons used Bonferroni-corrected Mann–Whitney U tests (see text). Values are observed scores on study-specific self-report measures. Because configural, metric, and scalar invariance were not tested across roles, differences in the preliminary mean index are not latent mean comparisons and may partly reflect item interpretation. These comparisons are hypothesis-generating. Post hoc factor-derived item means are only reported in Table S7. Bold *p* values indicate statistical significance (*p* < 0.05).

**Table 6:** Multivariable linear regression (heteroscedasticity-robust HC3 standard errors) of factors associated with the preliminary mean scenario-specific anxiety index (N = 400).

Variable	β (Robust SE)	95% CI	*p*
Female sex	0.55 (0.21)	0.15 to 0.96	**0.007**
Faculty member (ref: technician)	1.58 (0.55)	0.50 to 2.66	**0.004**
Specialist (ref: technician)	1.36 (0.28)	0.82 to 1.90	**<0.001**
Resident (ref: technician)	0.03 (0.26)	−0.48 to 0.53	0.913
Professional experience (per year)	−0.03 (0.02)	−0.06 to 0.01	0.126
≥6 critical incidents (ref: <6)	−0.64 (0.28)	−1.20 to −0.09	**0.024**
Overall burnout score (per point)	0.20 (0.06)	0.09 to 0.31	**<0.001**

β: unstandardized coefficient; SE: heteroscedasticity-robust (HC3) standard error; and CI: confidence interval. Covariates were entered simultaneously. Model R^2^ = 0.109 (adjusted R^2^ = 0.093); most variation remained unexplained. Measurement error in the study-specific outcome may reduce precision. Measurement error in predictors or nonclassical error can also bias coefficients and estimates for other covariates [26]. The coefficients are exploratory. The ≥6 vs. <6 term was an exploratory contrast arising from the questionnaire’s highest top-coded response category, not a validated or prespecified clinical threshold. The above-median sensitivity model (Table S4) does not define a clinical threshold. Bold *p* values indicate statistical significance (*p* < 0.05).

## Data Availability

Individual-level data are not publicly available because participants did not provide explicit consent for public sharing. A de-identified dataset may be available from the corresponding author upon reasonable request, subject to institutional and ethics requirements and an appropriate data-sharing arrangement. Cleaned analysis code is available from the corresponding author upon reasonable request.

## Supplementary Materials

The following supporting information can be downloaded at: https://www.mdpi.com/article/10.3390/healthcare14172815/s1, Table S1: STROBE checklist [23]; Table S2: CHERRIES checklist [24]; Table S3: 12-item scenario set (English translation and Turkish original); Tables S4–S6: Exploratory sensitivity and robustness models; Table S7: Post hoc, highly exploratory factor-derived item means; Table S8: False discovery rate sensitivity analysis of the six scenario-matched comparisons; Questionnaire S1: English questionnaire translation, including an English translation of the first-page consent screen; and Questionnaire S2: Original Turkish questionnaire, including the participant-facing first-page electronic consent screen.
