# Supplementary material for: Scenario-Related Anxiety, Self-Reported Critical Incidents, and Burnout Among Anesthesia Professionals: A Cross-Sectional Survey
Source: Healthcare (Basel). 2026 Sep 2;14(17):2815. doi: 10.3390/healthcare14172815 (PMC13565949; doi:10.3390/healthcare14172815)
Supplement: Supplementary file 1 [file healthcare-14-02815-s001.zip › healthcare-4470838-supplementary.pdf]

Supplementary Materials

Scenario-Related Anxiety, Self-Reported Critical Incidents, and Burnout among Anesthesia Professionals: A Cross-Sectional Survey

Table S1. STROBE Statement—checklist of items that should be included in reports of cross-sectional studies.

Item	No.	Recommendation	Location in manuscript
Title and abstract	1a	Indicate the study design with a commonly used term in the title or the abstract	Title; Abstract ("Cross-Sectional Survey")
	1b	Provide in the abstract an informative and balanced summary of what was done and what was found	Abstract
Background/rationale	2	Explain the scientific background and rationale for the investigation being reported	Section 1
Objectives	3	State specific objectives, including any prespecified hypotheses	Section 1, final paragraph
Study design	4	Present key elements of study design early in the paper	Section 2.1
Setting	5	Describe the setting, locations, and relevant dates, including periods of recruitment and data collection	Section 2.1 (Bursa Province, Türkiye; 20 June–6 July 2026; online survey)
Participants	6	Give the eligibility criteria, and the sources and methods of selection of participants	Section 2.2
Variables	7	Clearly define all outcomes, exposures, predictors, potential confounders, and effect modifiers	Sections 2.3 and 2.5
Data sources/ measurement	8	For each variable of interest, give sources of data and details of methods of assessment (measurement)	Section 2.3
Bias	9	Describe any efforts to address potential sources of bias	Sections 2.2 and 4.1 (one-response-per-account restriction; unknown invitation/view denominator; self-selection, recall, common method, measurement, and multiplicity limitations)
Study size	10	Explain how the study size was arrived at	Section 2.2 (target of 400 specified before recruitment in the ethics submission and study protocol; internal split-sample and multivariable uses and achieved-sample sensitivity calculation reported)
Quantitative variables	11	Explain how quantitative variables were handled in the analyses; describe which groupings were chosen and why	Section 2.5 (preliminary index; highest top-coded incident count category and exploratory ≥6-versus-<6 contrast; above-median sensitivity analysis)

Item	No.	Recommendation	Location in manuscript
Statistical methods	12a	Describe all statistical methods, including those used to control for confounding	Section 2.5 (continuous HC3 linear regression; complementary logistic model)
	12b	Describe any methods used to examine subgroups and interactions	Section 2.5 (role and sex comparisons)
	12c	Explain how missing data were addressed	Section 2.2 (no missing data by design: all items were required by the survey platform)
	12d	If applicable, describe analytical methods taking account of sampling strategy	Not applicable (convenience sample)
	12e	Describe any sensitivity analyses	Section 2.5 (ordinal incident count, complementary logistic and extended robustness models, selected family-wise Bonferroni corrections, and Benjamini–Hochberg FDR sensitivity analysis for the six scenario-matched comparisons)
Participants	13	Report numbers of individuals at each stage of the study	Sections 2.2 and 3.1 (400 complete submissions; view, participation, and completion rates not calculable)
Descriptive data	14	Give characteristics of study participants and information on exposures and potential confounders	Section 3.1, Table 1
Outcome data	15	Report numbers of outcome events or summary measures	Sections 3.3–3.7, Tables 3 and 4
Main results	16a	Give unadjusted estimates and, if applicable, confounder-adjusted estimates and their precision (e.g., 95% CI)	Section 3.8 and Table 6 (all adjusted β, robust SEs, 95% CIs, p values, R^2/adjusted R^2, and diagnostics); Tables S4–S6 (exploratory sensitivity and robustness models)
	16b	Report category boundaries when continuous variables were categorized	Section 2.5 (above-median preliminary mean scenario-specific anxiety index; sample median used only for a study-specific sensitivity categorization)
	16c	If relevant, consider translating estimates of relative risk into absolute risk	Not applicable
Other analyses	17	Report other analyses done—e.g., analyses of subgroups and interactions, and sensitivity analyses	Sections 3.2, 3.4, and 3.8; Tables S4–S8 (all explicitly labeled exploratory or hypothesis-generating)

Item	No.	Recommendation	Location in manuscript
Key results	18	Summarize key results with reference to study objectives	Section 4, first paragraph
Limitations	19	Discuss limitations of the study, taking into account sources of potential bias or imprecision	Section 4.1 (sampling/self-selection, measurement, invariance, common method, recall, clustering, non-equivalent items, and multiplicity)
Interpretation	20	Give a cautious overall interpretation of results considering objectives, limitations, multiplicity of analyses, results from similar studies, and other relevant evidence	Sections 4 and 5 (exploratory framing, modest R^2, and alternative explanations)
Generalizability	21	Discuss the generalizability (external validity) of the study results	Section 4.1
Funding	22	Give the source of funding and the role of the funders for the present study	Funding statement (no external funding)

STROBE: Strengthening the Reporting of Observational Studies in Epidemiology (von Elm et al., Lancet 2007;370:1453–1457).

Table S2. CHERRIES checklist (Checklist for Reporting Results of Internet E-Surveys).

Item category	Checklist item	Explanation	Reported in this study
Design	Describe survey design	Target population and sample frame	Cross-sectional survey of anesthesia professionals in Bursa Province; convenience sample recruited through professional messaging groups (Section 2.2)
IRB approval and informed consent	IRB approval	Ethics committee approval	Approved by the Medical Sciences Ethics Committee, University of Health Sciences, Bursa Yüksek İhtisas Training and Research Hospital (protocol code 2024-TBEK 2026/06-27; Section 2.4)
	Informed consent	Consent process and information provided	Electronic informed consent required before the questionnaire could be started (Section 2.4)
	Data protection	Protection of personal information	No directly identifying data were collected; account identifiers were not accessible to the investigators (Sections 2.2 and 2.4)
Development and pre-testing	Development and testing	Development and pre-testing of the instrument	Developed by the investigators based on clinical experience and a review of the relevant anesthesia complication literature; draft items were informally reviewed by anesthesiologists for clinical relevance and clarity and pilot-tested before data collection. No structured content validity procedure (e.g., Delphi process or content validity index) was performed (Sections 2.3 and 4.1).
Recruitment process	Open survey versus closed survey	Access restrictions	Invitation-based convenience web survey: the link was distributed within professional WhatsApp groups of anesthesia providers; because the link could be forwarded, strict access restriction could not be guaranteed (Section 2.2)
	Contact mode	Mode of first contact	Online invitation through professional messaging groups (Section 2.2)
	Advertising the survey	How the survey was announced	Invitation message in professional WhatsApp groups of anesthesia providers (Section 2.2)
Survey administration	Web/E-mail	Survey delivery mode	Web-based questionnaire (Google Forms; SSL-encrypted) (Section 2.2)
	Context	Website/community context	Professional messaging groups of anesthesia providers working in hospitals in Bursa Province (Sections 2.1 and 2.2)

Item category	Checklist item	Explanation	Reported in this study
	Mandatory/voluntary	Voluntariness	Participation was voluntary (Section 2.4)
	Incentives	Incentives offered	No incentives were offered
	Time/date	Data collection period	20 June–6 July 2026 (Section 2.1)
	Randomization of items or questionnaires	Randomization of items	Not performed; items were presented in a fixed order
	Adaptive questioning	Conditional display of items	Skip logic: post-event impact items (Section D) were displayed only to participants reporting a critical incident
	Number of items	Number of questionnaire items	52 items in six sections (Section 2.3)
	Number of screens (pages)	Questionnaire length in screens	Administered as a sectioned Google Forms questionnaire
	Completeness check	Consistency/completeness checks before submission	All items were required by the survey platform, so all submitted questionnaires were complete
	Review step	Ability to review and change answers	Respondents could navigate back and review responses before submission
Response rates	Unique site visitor	Definition of unique visitor	Not determinable; the form was limited to one response per signed-in account, but the invitation/view denominator was unknown (Section 2.2)
	View rate (ratio of unique survey visitors/unique site visitors)	View rate	Not calculable: the number of individuals who viewed the invitation could not be determined (Section 2.2)
	Participation rate (ratio of unique visitors who agreed to participate/unique first-survey-page visitors)	Participation rate	Not calculable (Section 2.2)
	Completion rate (ratio of users who finished the survey/users who agreed to participate)	Completion rate	All 400 submitted questionnaires were complete because responses were required; the number of individuals who opened but did not submit the survey is unknown, so a completion rate could not be calculated
Preventing multiple entries	Cookies used	Use of cookies	Not used for deduplication; response restriction via account login instead (Section 2.2)
	IP check	IP address checks	Not performed; limitation of multiple entries via one-response-per-account restriction (Section 2.2)
	Log file analysis	Log file analysis	Not performed

Item category	Checklist item	Explanation	Reported in this study
	Registration	User registration requirement	Responses were limited to one per signed-in account; multiple accounts could not be ruled out (Section 2.2)
Analysis	Handling of incomplete questionnaires	Whether incomplete questionnaires were analyzed	Not applicable: all submitted questionnaires were complete because responses were required
	Questionnaires submitted with an atypical timestamp	Timestamp screening	Not assessed
	Statistical correction	Weighting or propensity adjustment	No weighting or propensity adjustment. Bonferroni correction was applied within specified comparison families; Benjamini–Hochberg FDR was applied as a sensitivity analysis to the six scenario-matched comparisons. No study-wide multiplicity correction was applied.

CHERRIES: Checklist for Reporting Results of Internet E-Surveys (Eysenbach, J Med Internet Res 2004;6:e34).

Table S3. The 12-item scenario anxiety item set: English translation and Turkish original.

Instruction (English translation): "Please rate the level of anxiety that each of the following complication scenarios evokes in your anesthesia practice on a scale from 0 to 10 (0 = no anxiety at all, 10 = extreme anxiety)." Turkish original: "Aşağıdaki komplikasyon senaryolarının her birinin anestezi pratiğinizde size verdiği kaygı düzeyini 0–10 arasında işaretleyiniz (0 = Hiç kaygılanmıyorum, 10 = Son derece kaygılanıyorum)."

No.	Item (English translation)	Item (Turkish original)	Post hoc factor grouping used in Table S7
1	Difficult airway/failed intubation (CICO)	Zor havayolu/Entübasyon başarısızlığı (CICO)	F1
2	Intraoperative cardiac arrest	İntraoperatif kardiyak arrest	F1
3	Anaphylaxis	Anafilaksi	F1
4	Malignant hyperthermia	Malign hipertermi	F1
5	Intraoperative awareness	İntraoperatif farkındalık (awareness)	F2
6	Total spinal block	Total spinal blok	F1
7	Local anesthetic systemic toxicity (LAST)	Lokal anestezik sistemik toksisitesi (LAST)	F1
8	Massive hemorrhage/hemodynamic collapse	Masif kanama/Hemodinamik kollaps	F2
9	Medication error (wrong drug/dose)	İlaç hatası (yanlış ilaç/doz)	F1
10	Pediatric anesthesia complication	Pediatrik anestezi komplikasyonu	F1
11	Obstetric anesthesia emergency	Obstetrik anestezi acili	F2
12	Equipment failure	Ekipman arızası	F2

Each item is rated on an 11-point numeric response scale (0–10). The mean of all 12 items is a preliminary descriptive index, not a fully validated psychometric or clinical scale; it has no diagnostic cutoff and must not be used to diagnose or classify individuals or guide clinical decisions. The English translation has not been independently validated. Factor-derived item means are post hoc, highly exploratory summaries—not validated subscales—and should not support clinical or between-group inference (see Section 3.2).

Table S4. Complementary multivariable logistic regression of factors associated with an above-median preliminary mean scenario-specific anxiety index (sample median used only for sensitivity analysis; N = 400).

Variable	Adjusted OR	95% CI	p
Female sex	1.96	1.29–2.97	0.002
Faculty member (ref: technician)	4.89	1.82–13.16	0.002
Specialist (ref: technician)	2.76	1.58–4.82	<0.001
Resident (ref: technician)	1.11	0.63–1.95	0.719
Professional experience (per year)	0.99	0.96–1.02	0.518
≥6 critical incidents (ref: <6)	0.51	0.28–0.94	0.031
Overall burnout score (per point)	1.21	1.06–1.37	0.005

OR: odds ratio; CI: confidence interval. The above-median preliminary mean scenario-specific anxiety index is an exploratory, study-specific sensitivity categorization and not a validated clinical threshold or prediction endpoint. Hosmer–Lemeshow test: $\chi^2(8) = 3.74$, $p = 0.880$; Nagelkerke $R^2 = 0.120$; AUC = 0.670. This sensitivity model does not establish clinical importance.

Table S5. Extended robustness model: multivariable logistic regression of the above-median preliminary mean scenario-specific anxiety index additionally adjusted for institution type, weekly caseload, and main field of practice (N = 400).

Variable	Adjusted OR	95% CI	p
Female sex	1.87	1.22–2.87	0.004
Faculty member (ref: technician)	5.34	1.91–14.98	0.001
Specialist (ref: technician)	3.73	2.02–6.88	<0.001
Resident (ref: technician)	1.17	0.63–2.17	0.619
Professional experience (per year)	0.99	0.96–1.02	0.599
≥6 critical incidents (ref: <6)	0.52	0.28–0.97	0.039
Overall burnout score (per point)	1.19	1.04–1.36	0.010
University hospital (ref: training and research)	0.89	0.46–1.71	0.730
State hospital (ref: training and research)	0.57	0.27–1.20	0.139
Private hospital (ref: training and research)	0.39	0.15–1.01	0.054
Weekly anesthesia cases (per case)	0.97	0.96–0.99	0.002
Mixed/general practice (ref: cardiac or other subspecialty practice)	1.35	0.70–2.61	0.371

OR: odds ratio; CI: confidence interval. All covariates were entered simultaneously. This extended model is an exploratory robustness analysis; no study-wide multiplicity correction was applied.

Table S6. Extended robustness model for the continuous outcome: multivariable linear regression (heteroscedasticity-robust HC3 standard errors) of the preliminary mean scenario-specific anxiety index additionally adjusted for institution type, weekly caseload, and main field of practice (N = 400).

Variable	β (robust SE)	95% CI	p
Female sex	0.47 (0.20)	0.07 to 0.87	0.020
Faculty member (ref: technician)	1.71 (0.58)	0.56 to 2.85	0.003
Specialist (ref: technician)	1.61 (0.28)	1.05 to 2.16	<0.001
Resident (ref: technician)	0.09 (0.28)	−0.46 to 0.63	0.760
Professional experience (per year)	−0.02 (0.02)	−0.05 to 0.01	0.183
≥6 critical incidents (ref: <6)	−0.65 (0.29)	−1.21 to −0.08	0.024
Overall burnout score (per point)	0.18 (0.06)	0.07 to 0.30	0.001

Variable	β (robust SE)	95% CI	p
University hospital (ref: training and research)	−0.39 (0.33)	−1.05 to 0.26	0.240
State hospital (ref: training and research)	−0.45 (0.38)	−1.20 to 0.30	0.239
Private hospital (ref: training and research)	−1.17 (0.54)	−2.22 to −0.12	0.029
Weekly anesthesia cases (per case)	−0.026 (0.008)	−0.043 to −0.010	0.001
Mixed/general practice (ref: cardiac or other subspecialty practice)	0.39 (0.36)	−0.31 to 1.08	0.278

β: unstandardized coefficient; SE: heteroscedasticity-robust (HC3) standard error; and CI: confidence interval. All covariates were entered simultaneously; model $R^2 = 0.158$ (adjusted $R^2 = 0.132$). This extended model is exploratory, and coefficients may be affected by measurement error in the study-specific measures.

Table S7. Post hoc, highly exploratory factor-derived item means by professional role: median (IQR) and Kruskal–Wallis results (N = 400).

Variable	Faculty (n = 30)	Specialist (n = 108)	Resident (n = 117)	Technician (n = 145)	p *
Provisional Factor 1 item mean	7.00 (3.44-7.88)	7.00 (4.62-8.38)	5.88 (4.38-7.38)	5.25 (3.00-6.88)	<0.001
Provisional Factor 2 item mean	5.50 (3.38-6.69)	5.25 (3.75-6.50)	4.50 (2.75-6.25)	4.75 (3.00-6.25)	0.237

** Kruskal–Wallis test: provisional Factor 1 item mean, $H = 22.55$, $p < 0.001$; provisional Factor 2 item mean, $H = 4.23$, $p = 0.237$. Values are median (IQR). These are post hoc item groupings from a factor solution with inadequate confirmatory fit, not validated subscales or substantive domains. Configural, metric, and scalar invariance were not tested across roles. No study-wide multiplicity correction was applied. The results are purely hypothesis-generating.*

Table S8. Benjamini–Hochberg false discovery rate sensitivity analysis for the six scenario-matched comparisons (N = 400).

Scenario	Cited/not cited, n	Rating if cited, median (IQR)	Rating if not cited, median (IQR)	Raw p	BH-FDR q
Difficult airway	383/17	5 (2–8)	5 (3–7)	0.785	0.810
Cardiac arrest	223/177	7 (4–9)	5 (2–8)	0.002	0.012
Anaphylaxis	44/356	5.5 (3–7)	5 (3–8)	0.614	0.810
Malignant hyperthermia	8/392	9.5 (8–10)	7 (3–10)	0.049	0.146
Intraoperative awareness	16/384	3 (1.75–5)	4 (2–5)	0.495	0.810
Medication error	124/276	7 (4–9)	7 (4–10)	0.810	0.810

Two-sided asymptotic Mann–Whitney U tests compared scenario ratings between participants who did and did not cite the corresponding event among their two most serious career incident types. The Benjamini–Hochberg procedure was applied jointly to the six raw p values. q denotes the FDR-adjusted p value. Only the cardiac arrest comparison remained significant at $q < 0.05$. This family-specific analysis was exploratory and did not control multiplicity across the full study.

Questionnaire S1. Full study questionnaire (English translation of the Turkish original).

The survey was administered in Turkish (Google Forms). Questionnaire S1 is an English translation for readers and was not independently validated; its first section is an English translation of the participant-facing information-and-consent screen. Questionnaire S2 reproduces the original Turkish questionnaire and begins with the original mandatory consent screen. Only "Yes" permitted access; "No" ended the form. This ethical consent gate was not an eligibility criterion or one of the 52 analytic response variables. All displayed analytic items were required. Skip logic displayed C2–C6 and Section D only after C1 = "Yes". The participant-facing estimated completion time was 5–6 minutes.

Participant information and electronic informed consent (mandatory first screen)

Dear Participant,

You are invited to participate in a survey-based study entitled "Evaluation of Anesthesia Professionals' Attitudes and Experiences Regarding Feared Scenarios and Adverse Events Encountered in Anesthesia Practice."

This study is being conducted to identify the complication scenarios that anesthesia professionals fear most during clinical practice and to assess their attitudes and experiences regarding adverse events.

Your participation is entirely voluntary. You may leave the study at any time while completing the questionnaire. Your responses will be stored anonymously; your identity cannot be determined. Your decision to participate or not participate will not affect your professional status or institutional position. Your data will be used only for scientific purposes and will be reported only in aggregate. The questionnaire will take approximately 5–6 minutes to complete. This study is conducted by Specialist Physician Anıl ONUR. The study was approved by the Medical Sciences Ethics Committee of the University of Health Sciences, Bursa Yüksek İhtisas Training and Research Hospital on 10 June 2026 (decision no. 2024-TBEK 2026/06-27).

If you agree to participate in this study, please indicate your consent by selecting the box below.

Do you consent to participate in the study? * ☐ Yes ☐ No

Documentation note (not an analytic item): Only participants selecting "Yes" could access the questionnaire; selecting "No" ended the form. This note was added for documentation and was not displayed as a response item.

Section A — Sociodemographic and professional characteristics

A1. Your age (years): ______

A2. Your sex: ☐ Female ☐ Male ☐ Prefer not to say

A3. Your professional role: ☐ Anesthesiology and reanimation faculty member ☐ Anesthesiology and reanimation specialist ☐ Anesthesiology resident ☐ Anesthesia technician

A4. Total professional experience (years): ______

A5. Type of institution where you work: ☐ University hospital ☐ Training and research hospital ☐ State hospital ☐ Private hospital

A6. City of practice: (Bursa)

A7. Main field of practice: ☐ General surgical anesthesia ☐ Cardiac anesthesia ☐ Pediatric anesthesia ☐ Obstetric anesthesia ☐ Neuroanesthesia ☐ Intensive care ☐ Pain ☐ Mixed/general

A8. Average number of anesthesia cases per week: ______

Section B — Scenario-specific anxiety

B1. Please rate the level of anxiety that each of the following complication scenarios evokes in your anesthesia practice on a scale from 0 to 10 (0 = no anxiety at all, 10 = extreme anxiety). The twelve scenario items and their Turkish originals are listed in Table S3.

B2. Select the three scenarios that cause you the most anxiety, in order of importance (1 = most). Each rank was selected from a drop-down list of the twelve scenarios in B1.

B3. What is the main source of your anxiety in these scenarios? (Select the two most important) ☐ Possibility of permanent patient harm/death ☐ Medicolegal liability/lawsuit/investigation ☐ Fear of professional inadequacy/making an error ☐ Lack of experience or knowledge ☐ Inadequate team or institutional support ☐ Inadequate equipment/medication/infrastructure ☐ Workload/fatigue/burnout

Section C — Critical/adverse incident experience

C1. Have you encountered a serious/critical anesthesia complication or adverse event during your career? ☐ Yes ☐ No

Routing: if C1 = No, please proceed directly to Section E (Section D is only for those who have experienced an event); if C1 = Yes, continue with the questions below and Section D.

C2. How many times have you experienced such an event during your career? ☐ 1 ☐ 2–3 ☐ 4–5 ☐ 6 or more

C3. What type was the most serious event you experienced? (Select the two most serious event types) ☐ Difficult airway ☐ Cardiac arrest ☐ Anaphylaxis ☐ Malignant hyperthermia ☐ Awareness ☐ Medication error ☐ Other: ______

C4. Did the event result in permanent patient harm? ☐ Yes ☐ No ☐ I do not know

C5. Did you formally report the event? ☐ Yes ☐ No ☐ No such system exists in my institution

C6. Did this experience affect your clinical decision-making? ☐ Yes, positively ☐ Yes, negatively ☐ No

Section D — Post-event psychological impact (displayed only to participants reporting an incident)

D1. Thinking of the most serious event you experienced, to what extent do you agree with the following statements? (1 = strongly disagree, 2 = disagree, 3 = undecided, 4 = agree, 5 = strongly agree)

D1.1. I had difficulty putting what happened out of my mind. (1 2 3 4 5)

D1.2. My sleep or appetite was disturbed after the event. (1 2 3 4 5)

D1.3. I felt guilty or inadequate because of the event. (1 2 3 4 5)

D1.4. I felt anxious that a similar event would happen again. (1 2 3 4 5)

D1.5. My professional confidence was shaken after the event. (1 2 3 4 5)

D1.6. I received adequate support from my colleagues. (1 2 3 4 5)

D1.7. I received adequate support from my institution. (1 2 3 4 5)

D1.8. I considered leaving the profession because of this event. (1 2 3 4 5)

D2. Were you able to share this event with your colleagues? ☐ Yes ☐ No ☐ Partly

Section E — General burnout and work stress

E1. In general, how often do you experience the following in relation to your work? (1 = never, 2 = rarely, 3 = sometimes, 4 = often, 5 = always)

E1.1. I feel emotionally exhausted at work. (1 2 3 4 5)

E1.2. I feel overwhelmed by my workload. (1 2 3 4 5)

E1.3. I find it hard to go to work in the morning. (1 2 3 4 5)

E1.4. I experience work-related anxiety/tension. (1 2 3 4 5)

E1.5. I feel that the work I do is meaningful. (1 2 3 4 5)

E2. Overall, how burned out do you feel? (0 = not at all, 10 = extremely) 0 1 2 3 4 5 6 7 8 9 10

Section F — Institutional preparedness

F1. Does your institution have a standardized difficult airway protocol? ☐ Yes ☐ No ☐ I do not know

F2. Have you received simulation-based emergency scenario training in the past year? ☐ Yes ☐ No

F3. Is the adverse event reporting system in your institution functional? ☐ Yes ☐ No ☐ Partly

F4. In which areas do you think training/simulation is needed? (Select the two most important) ☐ Difficult airway ☐ Cardiopulmonary resuscitation ☐ Anaphylaxis management ☐ Medication safety ☐ Team communication ☐ Malignant hyperthermia

Thank you for your participation.

Scoring. The mean of the twelve B1 items (0–10) is used only as a preliminary descriptive scenario-specific anxiety index; it is not a validated psychometric or clinical scale, has no diagnostic cutoff, and must not be used for individual classification or clinical decisions. Provisional factor-derived item means are post hoc, highly exploratory summaries, not validated subscales. The post-event distress score is the mean of D1.1–D1.5 and D1.8 (1–5); D1.6 and D1.7 are analyzed separately. The burnout score is the mean of E1.1–E1.4 (1–5); E1.5 is analyzed separately, and E2 is a study-specific 0–10 rating. None of the study-specific composite scores has a clinical threshold or diagnostic interpretation. B2, B3, C1–C6, D2, and F1–F4 are analyzed descriptively.

Questionnaire S2. Full study questionnaire (original Turkish form).

ANKET FORMU — Anestezi Profesyonellerinin Anestezi Uygulamalarında Karşılaştıkları Korkulan Senaryolar ve İstenmeyen Olaylara İlişkin Tutum ve Deneyimlerinin Değerlendirilmesi. Katılımcıya gösterilen tahmini tamamlama süresi: 5–6 dakika.

Katılımcı bilgilendirme ve elektronik gönüllü onam (anket sorularından önce gösterilen zorunlu ilk ekran)

Sayın Katılımcı,

Sizi yürütülmekte olan "Anestezi Profesyonellerinin Anestezi Uygulamalarında Karşılaştıkları Korkulan Senaryolar ve İstenmeyen Olaylara İlişkin Tutum ve Deneyimlerinin Değerlendirilmesi" başlıklı ankete dayalı bir araştırmaya davet ediyoruz.

Bu araştırma anestezi profesyonellerinin klinik uygulamalar sırasında en çok korktukları komplikasyon senaryoları ve istenmeyen olaylara ilişkin tutum ve deneyimleri belirlemek amacıyla yürütülmektedir.

Katılımınız tamamen gönüllülük esasına dayanmaktadır. Anketin herhangi bir anında çalışmadan çıkabilirsiniz. Yanıtlarınız tamamen anonim şekilde saklanacaktır; kişisel kimliğiniz hiçbir şekilde tespit edilemez. Katılım yada katılmama kararınız mesleki statünüzü veya kurumsal kimliğinizi etkilemeyecektir. Verileriniz yalnızca bilimsel amaçla kullanılacaktır. Sadece grup bazında raporlanacaktır. Anketi tamamlamak yalnızca 5–6 dakika sürecektir. Bu çalışma Uz. Dr. Anıl ONUR tarafından yürütülmektedir. Çalışmamız S.B.Ü Bursa Yüksek İhtisas Eğitim ve Araştırma Hastanesi Tıp Bilimleri Etik Kurulu tarafından 10.06.2026 tarihinde 2024-TBEK 2026/06-27 karar numarasıyla onaylanmıştır.

Bu araştırmaya katılmayı kabul ediyorsanız lütfen aşağıdaki kutucuğu işaretleyerek onam verdiğinizi belirtiniz.

** Zorunlu soruyu belirtir*

Çalışmaya katılmayı onaylıyor musunuz? * ☐ Evet ☐ Hayır

Belgeleme notu (analitik madde değildir): Yalnızca 'Evet' seçeneğini işaretleyen katılımcılar ankete erişebilmiş, 'Hayır' seçeneği formu sonlandırmıştır. Bu açıklama belgeleme amacıyla eklenmiştir ve katılımcıya yanıt maddesi olarak gösterilmemiştir.

BÖLÜM A — Sosyodemografik Bilgiler

A1. Yaşınız (yıl): ______

A2. Cinsiyetiniz: ☐ Kadın ☐ Erkek ☐ Belirtmek istemiyorum

A3. Mesleki unvanınız: ☐ Anestezi ve Reanimasyon Akademisyeni (Öğretim Üyesi) ☐ Anestezi ve Reanimasyon Uzmanı ☐ Anestezi Asistanı (Araştırma Görevlisi) ☐ Anestezi Teknikeri / Teknisyeni

A4. Toplam mesleki deneyim süreniz (yıl): ______

A5. Çalıştığınız kurum türü: ☐ Üniversite Hastanesi ☐ Eğitim-Araştırma Hastanesi ☐ Devlet Hastanesi ☐ Özel Hastane

A6. Çalıştığınız şehir: (Bursa)

A7. Asıl çalışma alanınız: ☐ Genel cerrahi anestezisi ☐ Kardiyak anestezi ☐ Pediatrik anestezi ☐ Obstetrik anestezi ☐ Nöroanestezi ☐ Yoğun bakım ☐ Ağrı ☐ Karma / Genel

A8. Haftalık ortalama anestezi vaka sayınız: ______

BÖLÜM B — Senaryoya Özgü Kaygı Değerlendirmesi

B1. Aşağıdaki komplikasyon senaryolarının her birinin anestezi pratiğinizde size verdiği kaygı düzeyini 0–10 arasında işaretleyiniz. (0 = Hiç kaygılanmıyorum, 10 = Son derece kaygılanıyorum)

1. Zor havayolu / Entübasyon başarısızlığı (CICO) (0 1 2 3 4 5 6 7 8 9 10)
2. İntraoperatif kardiyak arrest (0 1 2 3 4 5 6 7 8 9 10)
3. Anafilaksi (0 1 2 3 4 5 6 7 8 9 10)
4. Malign hipertermi (0 1 2 3 4 5 6 7 8 9 10)
5. İntraoperatif farkındalık (awareness) (0 1 2 3 4 5 6 7 8 9 10)
6. Total spinal blok (0 1 2 3 4 5 6 7 8 9 10)
7. Lokal anestezik sistemik toksisitesi (LAST) (0 1 2 3 4 5 6 7 8 9 10)
8. Masif kanama / Hemodinamik kollaps (0 1 2 3 4 5 6 7 8 9 10)

9. İlaç hatası (yanlış ilaç / doz) (0 1 2 3 4 5 6 7 8 9 10)

10. Pediatrik anestezi komplikasyonu (0 1 2 3 4 5 6 7 8 9 10)

11. Obstetrik anestezi acili (0 1 2 3 4 5 6 7 8 9 10)

12. Ekipman arızası (0 1 2 3 4 5 6 7 8 9 10)

B2. En çok kaygılandıran 3 senaryoyu önem sırasıyla seçiniz (1 = en çok). Her sıra, B1'deki on iki senaryoluk açılır listeden seçilmiştir.

B3. Bu senaryolarda kaygınızın temel kaynağı nedir? (En önemli 2) ☐ Hastada kalıcı zarar / ölüm olasılığı ☐ Tıbbi-yasal sorumluluk / dava / soruşturma kaygısı ☐ Mesleki yetersizlik / hata yapma kaygısı ☐ Deneyim veya bilgi eksikliği ☐ Ekip veya kurumsal destek yetersizliği ☐ Ekipman / ilaç / altyapı yetersizliği ☐ İş yükü / yorgunluk / tükenmişlik

BÖLÜM C — Kritik / İstenmeyen Olay Deneyimi

C1. Kariyeriniz boyunca ciddi/kritik bir anestezi komplikasyonu veya istenmeyen olayla karşılaştınız mı? ☐ Evet ☐ Hayır

YÖNLENDİRME (skip logic): C1 = HAYIR ise lütfen doğrudan BÖLÜM E'ye geçiniz (Bölüm D yalnızca olay yaşayanlar içindir); C1 = EVET ise aşağıdaki sorularla ve Bölüm D ile devam ediniz.

C2. Kariyeriniz boyunca kaç kez böyle bir olay yaşadınız? ☐ 1 ☐ 2–3 ☐ 4–5 ☐ 6 ve üzeri

C3. Yaşadığınız en ciddi olay ne türdeydi? (En ciddi iki olay türünü seçiniz) ☐ Zor havayolu ☐ Kardiyak arrest ☐ Anafilaksi ☐ Malign hipertermi ☐ Awareness ☐ İlaç hatası ☐ Diğer: ______

C4. Bu olay sonucunda hastada kalıcı hasar oldu mu? ☐ Evet ☐ Hayır ☐ Bilmiyorum

C5. Bu olayı resmî olarak bildirdiniz mi? ☐ Evet ☐ Hayır ☐ Kurumda böyle bir sistem yok

C6. Bu deneyim klinik karar alma süreçlerinizi etkiledi mi? ☐ Evet, olumlu ☐ Evet, olumsuz ☐ Hayır

BÖLÜM D — Olay Sonrası Psikolojik Etkilenme (yalnızca C1 = Evet diyenler için)

D1. Yaşadığınız en ciddi olay sonrasında aşağıdaki ifadelere ne ölçüde katılıyorsunuz? (1 = Kesinlikle katılmıyorum, 2 = Katılmıyorum, 3 = Kararsızım, 4 = Katılıyorum, 5 = Kesinlikle katılıyorum)

D1.1. Olaydan sonra yaşananları zihnimden atmakta zorlandım. (1 2 3 4 5)

D1.2. Olay sonrası uyku düzenim veya iştahım bozuldu. (1 2 3 4 5)

D1.3. Olay nedeniyle kendimi suçlu ya da yetersiz hissettim. (1 2 3 4 5)

D1.4. Benzer bir olayın tekrar yaşanacağına dair kaygı duydum. (1 2 3 4 5)

D1.5. Olaydan sonra mesleki özgüvenim sarsıldı. (1 2 3 4 5)

D1.6. Meslektaşlarımdan yeterli destek gördüm. (1 2 3 4 5)

D1.7. Kurumumdan yeterli destek gördüm. (1 2 3 4 5)

D1.8. Bu olay nedeniyle mesleği bırakmayı düşündüm. (1 2 3 4 5)

D2. Bu olayı meslektaşlarınızla paylaşabildiniz mi? ☐ Evet ☐ Hayır ☐ Kısmen

BÖLÜM E — Genel Tükenmişlik ve İş Stresi

E1. Genel olarak işinizle ilgili aşağıdaki durumları ne sıklıkla yaşıyorsunuz? (1 = Hiçbir zaman, 2 = Nadiren, 3 = Bazen, 4 = Sıkça, 5 = Her zaman)

E1.1. İşte duygusal olarak tükenmiş hissediyorum. (1 2 3 4 5)

E1.2. İş yükümün altında ezildiğimi hissediyorum. (1 2 3 4 5)

E1.3. Sabah işe gitmek bana zor geliyor. (1 2 3 4 5)

E1.4. Mesleğimle ilgili kaygı/gerginlik yaşıyorum. (1 2 3 4 5)

E1.5. Yaptığım işin anlamlı olduğunu hissediyorum. (1 2 3 4 5)

E2. Genel olarak kendinizi ne ölçüde tükenmiş hissediyorsunuz? (0 = Hiç, 10 = Aşırı) 0 1 2 3 4 5 6 7 8 9 10

BÖLÜM F — Kurumsal Hazırlık

F1. Kurumunuzda zor havayolu için standardize bir protokol var mı? ☐ Evet ☐ Hayır ☐ Bilmiyorum

F2. Son 1 yılda simülasyon tabanlı acil senaryo eğitimi aldınız mı? ☐ Evet ☐ Hayır

F3. Kurumunuzda 'istenmeyen olay bildirimi' sistemi işlevsel mi? ☐ Evet ☐ Hayır ☐ Kısmen

F4. Hangi alanda eğitim/simülasyon gerektiğini düşünüyorsunuz? (En önemli 2) ☐ Zor havayolu ☐ Kardiyak resüsitasyon ☐ Anafilaksi yönetimi ☐ İlaç güvenliği ☐ Ekip iletişimi ☐ Malign hipertermi

Katılımınız için teşekkür ederiz.
